# Supplementary material for: Genome Sequencing and Analysis Reveal Potential High-Valued Metabolites Synthesized by Lasiodiplodia iranensis DWH-2
Source: J Fungi (Basel). 2023 Apr 28;9(5):522. doi: 10.3390/jof9050522 (PMC10219381; doi:10.3390/jof9050522)
Supplement: Supplementary file 1 [file jof-09-00522-s001.zip › jof-2298537-supplementary.pdf]

Genome Sequencing and Analysis reveal Potential High-Valued Metabolites Synthesized by *Lasiodiplodia iranensis* DWH-2

Ruiying Li, Pu Zheng,* Xingyun Sun, Wenhua Dong, Ziqiang Shen, Pengcheng Chen, Dan Wu

The Key Laboratory of Industrial Biotechnology, School of Biotechnology, Jiangnan University, Ministry of Education, Wuxi 214122, China

* Correspondence: zhengpu@jiangnan.edu.cn; Tel./Fax: +86-510-85918156

Supporting Information

Table. S1. Genomes of *Lasiodiplodia*.

Table. S2. Genome features of *L. iranensis*.

Table. S3. List of CAZy gene clusters existing in *L. theobromae*, which not in *L. iranensis* DWH-2.

Table. S4. Biosynthetic gene clusters (BGCs) of *L. iranensis*.

Figure. S1. Phylogenetic tree of strain DWH-2 based on ITS-rDNA. The PCR amplification of the ITS region using universal primers (ITS1, ITS4) of the template DNA of *L. iranensis* DWH-2. Sequence alignment was performed on NCBI, and it was found that DWH-2 has the closest genetic relationship with the strain *Lasiodiplodia iranensis isolate* CMW25232. It is preliminarily confirmed that DWH-2 was *Lasiodiplodia* sp. and was named *Lasodiplodia iranensis* DWH-2.

Figure. S2. α-Linolenic acid metabolism according to KEGG pathway. Found genes belonging to the α- Linolenic acid metabolism, and annotated genes in genome of *L. iranensis* DWH-2 are labeled on the metabolic map based on their expression enzymes with green mark. The route of JA biosynthesis was showed by red lines.

Table. S1. Genomes of *Lasiodiplodia*.

Assembly Accession	Organism	Strain	Size(Mb)	Assembly Submission Date
GCA_002111425.1	*Lasiodiplodia theobromae*	CSS-01s	43.28	2017/4/26
GCA_008079295.1	*Lasiodiplodia theobromae*	307	43.59	2019/8/23
GCA_008931885.1	*Lasiodiplodia theobromae*	LA-SOL3	43.92	2019/10/9
GCA_009801085.1	*Lasiodiplodia* sp. COLG20	COLG20	43.53	2019/12/27
GCA_009801105.1	*Lasiodiplodia* sp. COLG96	COLG96	43.15	2019/12/27
GCA_009829795.1	*Lasiodiplodia gonubiensis*	CBS 115812	41.14	2020/1/3
GCA_009829805.1	*Lasiodiplodia pseudotheobromae*	CBS 116459	43	2020/1/3
GCA_009829825.1	*Lasiodiplodia theobromae*	CBS 164.96	42.97	2020/1/3
GCA_012971845.1	*Lasiodiplodia theobromae*	AM2As	43.69	2020/5/4
GCA_018153875.1	*Lasiodiplodia theobromae*	A20-4	43.03	2021/4/28
GCA_023079305.1	*Lasiodiplodia sp. KE8409*	KE8409	43.98	2022/4/21
GCA_023086975.1	*Lasiodiplodia theobromae*	KE84463	51.87	2022/4/21
GCA_023087325.1	*Lasiodiplodia theobromae*	KE88424	43.47	2022/4/21
GCA_023087365.1	*Lasiodiplodia theobromae*	KE8366	44.35	2022/4/21
GCA_023087405.1	*Lasiodiplodia theobromae*	KE88423	43.24	2022/4/21
GCA_023087525.1	*Lasiodiplodia* sp. KE8368	KE8368	50.97	2022/4/21
GCA_023087545.1	*Lasiodiplodia pseudotheobromae*	KET9	45.88	2022/4/21
GCA_023089105.1	*Lasiodiplodia citricola*	KE87127	44.01	2022/4/21
GCA_023373445.1	*Lasiodiplodia theobromae*	JMB122	46.93	2022/4/20
This study	*Lasiodiplodia iranensi*s	DWH-2	43.01	

Table. S2 DNA libraries and sequencing data for *L. iranensis*.

Data type	Sequencing type	Insert size (bp)	Raw reads	Clean reads	Reads length (bp)
Illumina	Paired-end	250	31581636*2	23168619*2	150

Table. S3. List of CAZy gene clusters existing in *L. theobromae*, which not in *L. iranensis* DWH-2.

Cluster name	Protein number	Swissprot id	CAZy family
cluster570	3	N/A	AA11
cluster7	5	P12676	AA3
cluster564	3	Q7X2H8	AA3
cluster560	3	B5WWZ8	AA3_1
cluster533	3	A0A075TR33	AA7
cluster547	3	A0A0E0RTV6	AA7
cluster551	3	N/A	AA7
cluster554	3	Q4WLW6	AA7
cluster557	3	A0ST43	AA7
cluster562	3	A0ST43	AA7
cluster567	3	Q5KTN0	AA7
cluster572	3	G3XMD0	AA7
cluster549	3	Q00298	CE5
cluster545	3	N/A	GH106
cluster559	3	A0A024SMV2	GH109
cluster561	3	P28493	GH152
cluster568	3	P40954	GH18
cluster569	3	Q0CI48	GH2
cluster553	3	B8NMR5	GH3
cluster546	3	A7LXT8	GH43_14
cluster528	3	A2QT85	GH43_6
cluster556	3	Q9UKM7	GH47
cluster555	3	B8NKA3	GH51
cluster548	3	N/A	GH78
cluster552	3	A2QEQ6	GH79
cluster565	3	D4ATR3	GH92
cluster571	3	D4ATR3	GH92
cluster550	3	Q9XIG1	GT1
cluster563	3	Q9XIG1	GT1
cluster558	3	P87172	GT4
cluster575	3	Q5B7W0	GT50
cluster574	3	O43053	GT57
cluster566	3	N/A	GT69
cluster573	3	Q4HVS2	GT8

Table. S4. BCGs of *L. iranensis*.

Region	Type	Location	Most similar known cluster	Similarity
Region 1.1	terpene	1425937-1447070		
Region 2.1	NRPS[1],NRPS-like	2373909-2450413		
Region 3.1	T1PKS[2]	76530-123133	THN	100%
Region 3.2	NRPS-like	568316-611630		
Region 4.1	T1PKS	72108-109517	patulin	40%
Region 4.2	NRPS-like,NRPS	132459-197200	dimethylcoprogen	100%
Region 4.3	NRPS	247679-294874		
Region 4.4	T1PKS	407236-440499		
Region 4.5	T1PKS	1771288-1816893	(R)-Mellein	100%
Region 6.1	T1PKS	482092-574112		
Region 8.1	NRPS-like	792046-835362		
Region 8.2	terpene	1347213-1368584	brassicicene C	33%
Region 9.1	NRPS-like	1105388-1148713		
Region 12.1	NRPS,T1PKS	89207-169284		
Region 12.2	terpene	783627-805265		
Region 13.1	betalactone	554758-585325		
Region 14.1	terpene	274922-296204		
Region 14.2	NRPS	899827-956824		
Region 16.1	NRPS	936741-1037124		
Region 17.1	betalactone	51860-75567		
Region 18.1	terpene	491600-516721		
Region 20.1	terpene	661246-682593		
Region 21.1	NRPS-like	161018-204395		
Region 22.1	NRPS,T1PKS	65658-130700		
Region 22.2	NRPS	150776-196625		
Region 23.1	T1PKS	56262-104525		
Region 24.1	T1PKS,NRPS	359904-411908		
Region 24.2	NRPS	418113-469972	verticillin	38%
Region 26.1	NRPS-like	815-44517		
Region 28.1	NRPS-like	236580-276918		
Region 28.2	NRPS	396699-449235		
Region 30.1	NRPS	76716-130449		
Region 33.1	terpene	383977-406943		
Region 34.1	NRPS,T1PKS	330627-451859	phomasetin	42%
Region 35.1	NRPS	211237-265866		
Region 36.1	terpene	294651-315814		
Region 39.1	T1PKS	234316-279894	secalonic acids	37%
Region 40.1	NRPS	9442-55306		
Region 44.1	NRPS-like	31918-75729		
Region 45.1	T1PKS	188470-250354	lasiodiplodin	66%
Region 46.1	NRPS-like	2568-42835		
Region 46.2	T1PKS,NRPS-like	159456-210189		

Region 47.1	T1PKS,NRPS-like	168332-204789
Region 52.1	NRPS-like	53304-96603
Region 53.1	T1PKS,terpene	28121-74160
Region 64.1	terpene	1-19729
Region 70.1	T1PKS	1-30304

[1]NRPS: non-ribosomal peptide synthase.

[2]T1PKS: Type I Poly Keytide Synthase.

Figure. S1.

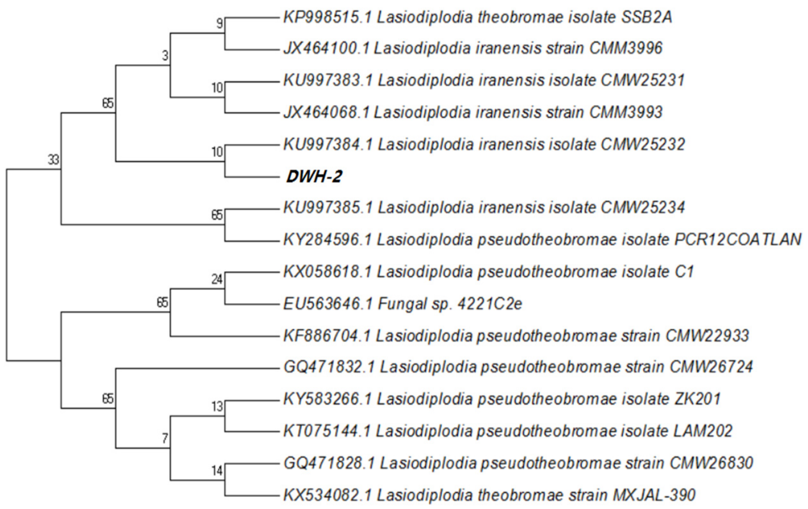
KP998515.1 Lasiodiplodia theobromae isolate SSB2A
JX464100.1 Lasiodiplodia iranensis strain CMM3996
KU997383.1 Lasiodiplodia iranensis isolate CMW25231
JX464068.1 Lasiodiplodia iranensis strain CMM3993
KU997384.1 Lasiodiplodia iranensis isolate CMW25232
DWH-2
KU997385.1 Lasiodiplodia iranensis isolate CMW25234
KY284596.1 Lasiodiplodia pseudotheobromae isolate PCR12COATLAN
KX058618.1 Lasiodiplodia pseudotheobromae isolate C1
EU563646.1 Fungal sp. 4221C2e
KF886704.1 Lasiodiplodia pseudotheobromae strain CMW22933
GQ471832.1 Lasiodiplodia pseudotheobromae strain CMW26724
KY583266.1 Lasiodiplodia pseudotheobromae isolate ZK201
KT075144.1 Lasiodiplodia pseudotheobromae isolate LAM202
GQ471828.1 Lasiodiplodia pseudotheobromae strain CMW26830
KX534082.1 Lasiodiplodia theobromae strain MXJAL-390
9
3
10
65
10
33
65
24
65
65
13
7
14

Figure. S2.

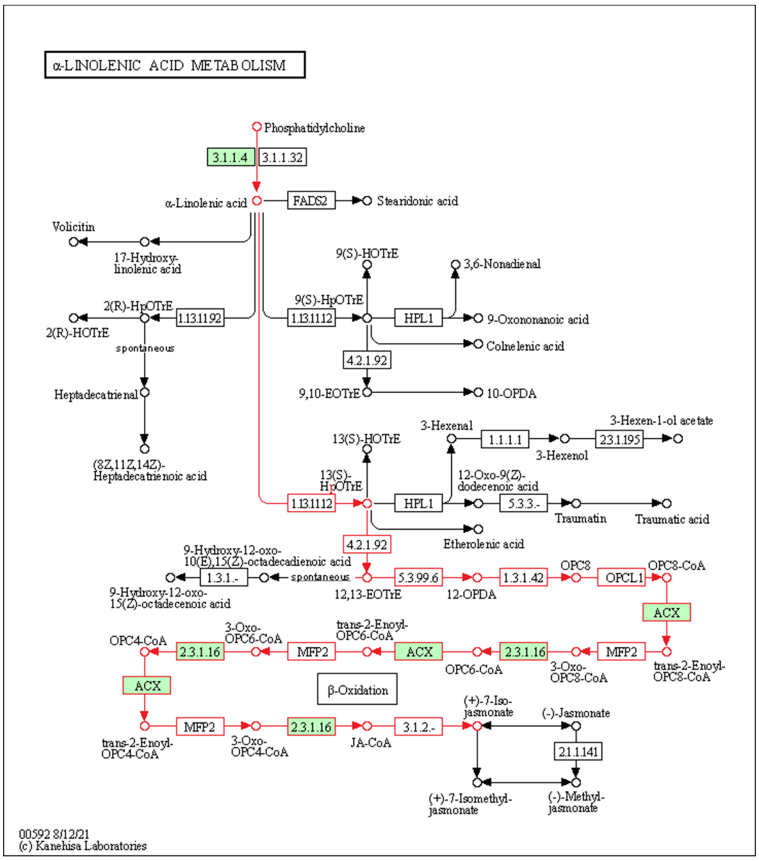

α-LINOLENIC ACID METABOLISM
Phosphatidylcholine
3.1.1.4
3.1.1.32
α-Linolenic acid
FADS2
Stearidonic acid
Volicitin
17-Hydroxy-linolenic acid
9(S)-HOTrE
3,6-Nonadienal
2(R)-HpOTrE
1.13.11.92
2(R)-HOTrE
9(S)-HpOTrE
1.13.11.12
HPL1
9-Oxononanoic acid
Colnelenic acid
spontaneous
4.2.1.92
Heptadecatrienal
9,10-EOTrE
10-OPDA
3-Hexenal
3-Hexen-1-ol acetate
1.1.1.1
2.3.1.195
13(S)-HOTrE
3-Hexenol
(8Z,11Z,14Z)-Heptadecatrienoic acid
13(S)-HpOTrE
12-Oxo-9(Z)-dodecenoic acid
1.13.11.12
HPL1
5.3.3.-
Traumatin
Traumatic acid
Etherolenic acid
4.2.1.92
9-Hydroxy-12-oxo-10(E),15(Z)-octadecadienoic acid
OPC8
OPC8-CoA
1.3.1.-
spontaneous
5.3.99.6
1.3.1.42
OPCL1
9-Hydroxy-12-oxo-15(Z)-octadecenoic acid
12,13-EOTrE
12-OPDA
ACX
3-Oxo-OPC6-CoA
trans-2-Enoyl-OPC6-CoA
OPC4-CoA
2.3.1.16
MFP2
ACX
2.3.1.16
MFP2
OPC6-CoA
3-Oxo-OPC8-CoA
trans-2-Enoyl-OPC8-CoA
ACX
β-Oxidation
(+)-7-Iso-jasmonate
(-)-Jasmonate
MFP2
2.3.1.16
3.1.2.-
2.1.1.141
trans-2-Enoyl-OPC4-CoA
3-Oxo-OPC4-CoA
JA-CoA
(+)-7-Isomethyl-jasmonate
(-)-Methyl-jasmonate
00592 8/12/21
(c) Kanehisa Laboratories
